# Validity and Reliability of Accelerometer-Based Gait Assessment in Patients with Diabetes on Challenging Surfaces

**DOI:** 10.1155/2012/954378

**Published:** 2012-07-31

**Authors:** Eling D. de Bruin, Michèle Hubli, Pamela Hofer, Peter Wolf, Kurt Murer, Wiebren Zijlstra

**Affiliations:** ^1^Institute of Human Movement Sciences and Sport, ETH, 8093 Zurich, Switzerland; ^2^Sensory-Motor Systems Lab, ETH, Zurich, Switzerland; ^3^Center for Human Movement Sciences, University Medical Center Groningen, University of Groningen, Groningen, The Netherlands; ^4^Institut für Bewegungs- und Sportgerontologie, Deutsche Sporthochschule Köln, Köln, Germany

## Abstract

Walking on irregular terrain influences gait of diabetic patients. We investigate the test-retest reliability and construct validity of gait measured with the DynaPort MiniMod under single and dual task conditions in diabetic patients walking on irregular terrain to identify the measurement error (precision) and minimal clinical detectable change. 29 patients with Type 2 diabetes were measured once, and 25 repeated the measurement within 7 days. Patients walked on a therapy garden walkway. Differences between three groups of diabetics with various levels of lower extremity neuropathy were analyzed with planned contrasts. ICC was excellent for intervisit measurements with ICC's >0.824. Bland and Altman Plots, SEM, and SDD showed precise values, distributed around zero for both test conditions. A significant effect of grouping on step length performance hints at possible construct validity of the device. Good reliability of DynaPort MiniMod measurements on a therapy garden walkway and an indication for discriminatory capability suggests that DynaPort MiniMod could facilitate the study of gait in diabetic patients in conditions close to real-life situations. Good reliability, small measurement error, and values of minimal clinical detectable change recommend the further utilization of DynaPort MiniMod for the evaluation of gait parameters in diabetic patients.

## 1. Introduction

The World Health Organization has described type 2 diabetes as an international epidemic. Current estimates suggest that the number of persons with diabetes will reach 300 million by 2025 [1]. Fifty percent of patients who have diabetes for more than 20 years develop peripheral neuropathy (PN), which affects nerve function from the periphery to more proximal regions [2, 3]. Because the increasing prevalence of diabetes is accompanied by gait problems and a heightened risk of falling, there is an increased need for understanding the possible gait pattern changes diabetic patients are confronted with [4]. It has, furthermore, been demonstrated that patients with diabetes may also improve their gait due to specific exercise programs [5, 6].

In this context gait analysis is usually performed in specialized kinesiology laboratories. Cameras, force platforms, and magnetic and ultrasound systems are thereby often used technologies for the gait analysis [7, 8]. However, time expenditure and financial constraints limit their use in clinical practice [9]. Moreover, gait analyses are traditionally performed indoors, on a predefined, clean, and flat specific pathway. Such conditions enable precise recording but are not representative of the real-life context. Activities of daily life require us to move about in challenging environments and to walk on varied surfaces. Irregular terrain has been shown to influence gait parameters such as speed, especially in a population at risk for falling [10], for example, patients with Diabetes [11, 12]. Furthermore, the fact that falling mainly occurs in a complex environment [13] under attention demanding conditions emphasizes the need for clinicians to objectively record gait data in a real-life context [14] under dual task conditions [15].

The recent use of body-fixed sensors suggests that they could serve as a tool for analyzing the gait of patients in more challenging walking environments [16–18]. In comparison with other motion measurement devices, body-fixed sensors have the advantage of being lightweight and portable, which enables subjects to move relatively freely. They permit data collection in a challenging environment; they are easy to use, provide a good ratio in terms of cost and amount of information retrieved, and can capture data from many gait cycles. Thus they seem ideal for extending our understanding of gait changes in specific populations by performing measures in real-life conditions, for example, in diabetic patients [19]. An objective evaluation in real-life conditions might help understand the causes of diabetic gait problems and ultimately facilitate the choice or the development of appropriate physical treatment. Therefore, the potential of body-fixed sensor approaches should be investigated in the diabetic population in order to ensure the validity and the reliability of data recorded during gait analysis under single and dual task conditions on changing types of surfaces.

To be clinically useful, an assessment procedure must have a small measurement error to detect a real change and must be able to distinguish between subpopulations for example, diabetic patients with and without various stages of peripheral neuropathy. A test-retest difference in a patient with a value smaller than the standard error of the measurement (SEM) is likely to be the result of “measurement noise” and is unlikely to be detected reliably in practice; a difference greater than the smallest real difference is highly likely (with 95% confidence) to be a real difference [20]. Another example of these statistics is the smallest detectable difference (SDD) [21]. The DynaPort MiniMod body-fixed sensor has previously been shown to be reliable, valid, and valuable in elderly for the analysis of gait performed on challenging surfaces [22–25]. To date, little is known about the variability in gait measures within the diabetic population and the reliable use of accelerometers in these patients. With this in mind, we conducted this study to (a) investigate the validity and reliability of gait parameters measured with DynaPort MiniMod in diabetic patients walking under single and dual task conditions on a challenging walking course, (b) identify the measurement error (precision), and (c) identify the smallest clinical detectable difference. We hypothesized [1] that walking quality in patients with diabetes can be reliably measured with accelerometers [2], that the walking quality is different in patient subgroups (we expect diabetic neuropathy to change gait quality compared to the group with no neuropathy), and [3] we believe that severe neuropathy effects walking quality more than mild neuropathy.

## 2. Methods

The study was approved by the ethics committee in Canton Zurich. All participants received written and oral information and were requested to sign an informed consent statement.

### 2.1. Subjects

A convenience sample of 31 patients with diabetes Type 2 (with and without neuropathy) was recruited from the patients consulting the Division of Endocrinology, Diabetes and Clinical Nutrition, University Hospital of Zurich (Table 1). Patients were included if they were medically diagnosed with diabetes Type 2, were between 50 and 70 years of age, and had the ability to walk without assistive devices. Patients were excluded if they had concomitant foot ulcer, orthopaedic or surgical problems influencing gait parameters, a nondiabetic neuropathy (due to Charcot-Marie-Tooth disease, alcohol, or thyroid dysfunction), or neurological pathology influencing gait parameters.

Before gait analysis started, patients were assigned to one of three groups: “DIABETIC,” “MILD NEUROPATHY,” and “SEVERE NEUROPATHY” based on three tests. A Neurometer CPT electrodiagnostic device was used for sensory nerve conduction threshold (sNCT) evaluations at the great toe by determining current perception threshold (CPT) levels. CPT permits diagnosis of neuropathy due to its ability to diagnose and quantify hyperaesthesia [26]. The used Rapid Screening CPT (R-CPT) resulted in a value between 1 and 25, where the higher numbers indicate worse nerve conduction. The value was used to grade neuropathy: no neuropathy = 6–13, moderate neuropathy = 14–19, severe neuropathy = 20–25. The Rydel-Seiffer tuning fork test was used to assess the vibratory threshold perception at the base of the great toe as a good predictor for impairment of the vibratory senses and, therefore, also usable to diagnose neuropathy [27–29]. The Rydel-Seiffer tuning fork test acquires values between 0 and 8, where the higher values indicate better vibratory senses. Patients were grouped by the test in one of the three categories with application of an age-related correction [30]. The third test used was the Semmes-Weinstein monofilament test, a good test to diagnose but not to quantify neuropathy [31]. If the subjects did not notice five of seven stimuli, a neuropathy was diagnosed.

Testing and group assignment was performed by an MD unfamiliar with the study design and the patients. Based on the results of all three tests the MD categorized the patients in one of three categories. The MD principally considered the results from the Neurometer CPT/C tests where three values for every frequency were obtained for the right and the left great toe. If at least two frequencies of the worse foot had a value over 14, the subject was allocated to the “moderate neuropathy” group. If at least two frequencies of the worse foot had a value over 19, the subject was allocated to the “severe neuropathy” group. If there were any uncertainties in the group allocation according to R-CPT values, the Rydel-Seiffer tuning fork test was the next criteria considered. The loading of the group arrangement's criteria was Neurometer CPT/C > Rydel-Seiffer tuning fork > Semmes-Weinstein monofilament test.

After the analysis of nerve conduction the gait analysis started.

### 2.2. Apparatus

A triaxial accelerometer (DynaPort MiniMod, McRoberts BV, The Hague, The Netherlands) was used to measure pelvic accelerations. The accelerometer was placed at the lower back of the subject with the center of the device at the level of the second sacral vertebrae.

### 2.3. Test Procedures

**Figure 1:**
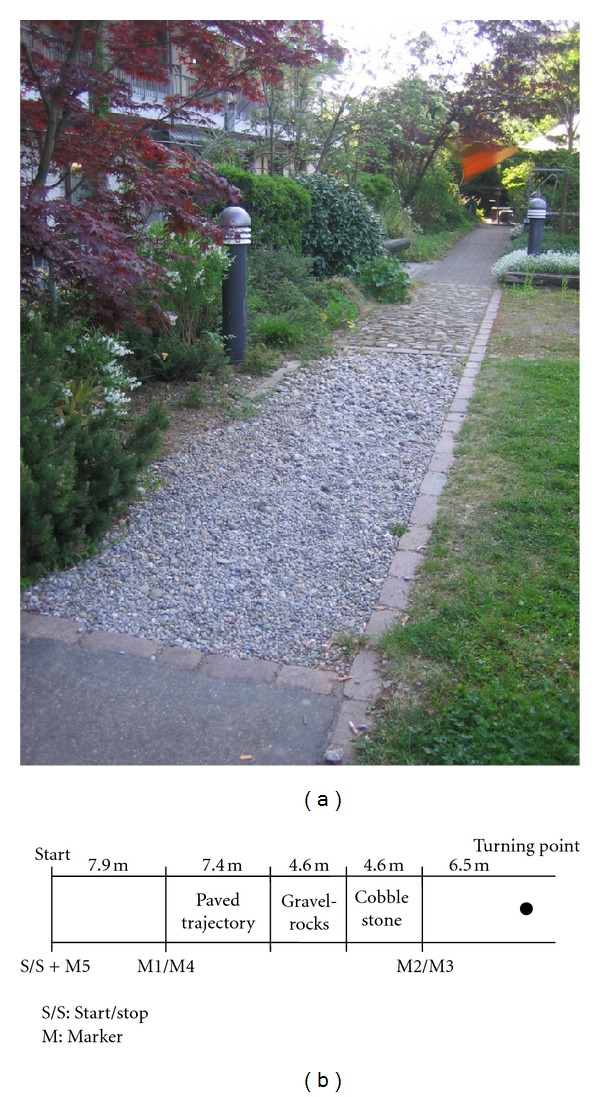
(a) The gait garden walkway with paved trajectory, cobble stones, and gravel rocks. (b) Schematic representation of the walkway and test procedure used. M signifies markers that are set in the signal to recognize the gait data for analyses.

Each subject was assessed during usual walking at preferred velocity under two different conditions over an outdoor gait therapy walkway with different surfaces: (1) silent walking on the walkway and (2) walking on the walkway with a counting task. The walkway contained a paved trajectory, cobble stones, and gravel rocks (Figure 1(a)). The complete walkway was 31 meter long and 1.5 meter wide. To measure steady state- walking, the 16.6 meters (with the three different surfaces) of the walking course was used as the test distance. The remaining parts of the walkway were used for acceleration and deceleration. At the end of the first 31 meters the subjects had to stop for two seconds, then turn around, and walk the walkway back to the starting point. At the beginning the measurement was started (S), and, at the end, the measurement was stopped (S + M5; Figure 1(b)).

1. Test run with subject's preferred walking speed: the subject received the most important information: no speaking, hold arms out of the pocket, and try not to stop walking during the measurement.
2. First trial with preferred walking speed: the subject was briefed to “*walk like you would bring a letter to the mailbox*” (single task).
3. Second trial with preferred walking speed and an additional cognitive task (count backwards aloud in steps of three): the subject had to walk and count aloud in steps of three. The subject was briefed to “*try to walk and count at the same time. Do not favour one task over the other but try to perform these concurrently*” (dual task).

The dual task was subtracting repeatedly the number three starting from 200 down and was practiced before gait testing while sitting on a chair. Subjects were told to try and perform both tasks at the same time without prioritizing either the walking or counting. A small receiver was mounted on the accelerometer and the researcher placed a marker in the data through triggering by the use of a remote control when the subjects passed distance lines (Figure 1(b)). The researcher walked alongside the subjects to ensure their safety. At the end of the last trial the SD card was removed from the accelerometer, and the measurements were checked for completeness on a laptop. The subject was asked to come again for the retest one week later at the same time and to wear the same shoes as during the first trial.

Per trial, all measured data between the two trigger signals (M1-M2/M3-M4) were used for analysis. Walking speed (*V*), cadence, mean values ($\overset{-}{X}$) of step duration (SDu) and step length (SL), and corresponding standard deviations (SD) were calculated for each subject and each trial.

## 3. Statistical Analyses 

Normality of the data was tested with the Kolmogorov-Smirnov test. Descriptive statistics were used to define the study population and to calculate gait characteristics.

We used the intraclass correlation (ICC_(2,1)_) with 95% confidence intervals to calculate intervisit reliability between visit 1 and visit 2. ICC_(2,1)_ was used because individual ratings constitute the unit of analysis, and raters and subjects were conceived as being a random selection. There was one week between visit 1 and 2. To interpret ICC_(2,1)_ values we used benchmarks suggested by Shrout and Fleiss [32] (>0.75 excellent reliability, 0.4–0.75 fair to good reliability, and <0.4 poor reliability). To evaluate precision the 95% limits of agreement statistics (Bland and Altman) were used. It expresses the degree of error proportional to the mean, and was calculated as $\overset{-}{d}\pm 2{\text{SD}}_{\text{diff}}$ [33, 34], where $\overset{-}{d}$ is the mean of the difference and SD_diff_ the standard deviation of the difference. The measurement error (standard error of the mean difference (SEM)) was reported, and the smallest detectable difference (SDD) for each parameter was calculated as described by de Vet et al. [35]. SEM was derived by $\sigma \sqrt{\left(1-\text{ICC}\right)}$ in which *σ* represents the total variance [36]. The smallest detectable change was calculated with the formula $1.96\times \text{SEM}\times \sqrt{2}$.

To identify differences between groups we used an analysis with planned contrasts [37]. All statistical analyses were performed using SPSS 17 for Windows (SPSS Inc.).

## 4. Results

Of the 31 patients screened for eligibility all met the inclusion criteria. Data from two individuals were, however, not available. One person presented with hypersensibility of the feet and could not be measured. Technical problems prevented data acquisition for the second person. This resulted in complete data for 29 patients (21 male and 8 female) at baseline, mean age: 61.9 (±5.5) years; body mass index: 28.2 (±3.5)) kg/m^2^; leg length 0.84 (±0.06) m (Table 1).

Twelve patients were categorized as “DIABETIC,” eleven as “MILD NEUROPATHY,” and six as “SEVERE NEUROPATHY.” Post hoc ANOVA revealed that the groups did not differ in Age *F*(2, 26) = 0.949, *P* = .40; height *F*(2, 26) = 1.26, *P* = .302; SDu *F*(2, 26) = 1.99, *P* = .157; *VF*(2, 26) = 3.01, *P* = .067; cadence *F*(2, 26) = 1.98, *P* = .159 and showed to be different for weight *F*(2, 26) = 4.729, *P* = .018; BMI *F*(2, 26) = 4.28, *P* = .025; SL *F*(2, 26) = 3.14, *P* = .048.

Four patients were unable or refused to perform the retest due to time limitations or lack of motivation. For the reliability testing we had twenty-five patients performing retesting (17 male and 8 female); mean age: 61 (±5.7) years; Body Mass Index: 28.7 (±3.5) kg/m^2^, leg length 0.83 (±0.06) m. Eleven patients were “DIABETIC,” eight “MILD NEUROPATHY” and six “SEVERE NEUROPATHY”.

### 4.1. Differences between the Walking Conditions

Table 2 presents means and SDs of both tests. Significant differences between the two test conditions at baseline, single versus dual task walking, were identified for all gait parameters (walking speed: *t*(28) = 3.616, *P* = .001, cadence: *t*(28) = 3.221, *P* = .003, step duration: *t*(28) = −3.112, *P* = .004, and step length: *t*(28) = 2.308, *P* = .029. Walking speed, step length, and cadence were significantly decreased under dual tasking, and step duration was significantly increased compared to normal walking.

### 4.2. Reliability

All data were normally distributed and showed no heteroscedasticity. The results of the repeated measurements for the different gait parameters SDu, SL, *V*, and Cadence are presented in Table 2. Except for cadence under dual task condition there were no differences in walking between visit 1 and 2.

**Figure 2:**
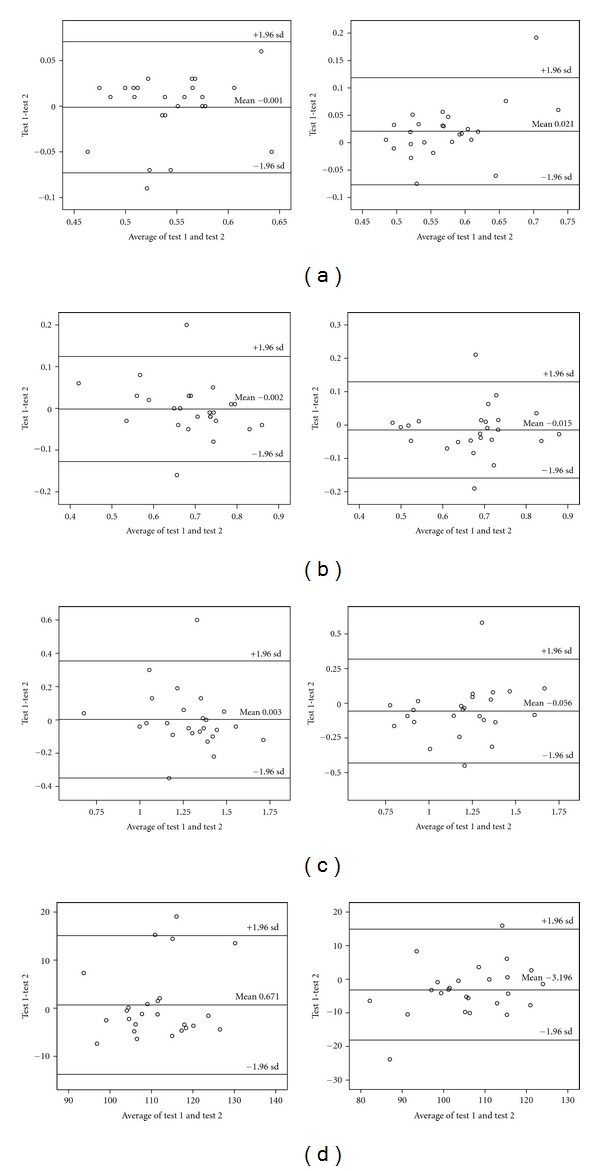
Bland-Altman plots of (a) step duration, (b) step length, (c) walking velocity, and (d) cadence (top to bottom). Left side represents the single task condition, and the right side represents the dual task walking.

All gait parameters on the walking trajectory under single and dual task walking with regard to test retest reliability are illustrated in Figure 2 by Bland-Altman plots. The results of the test retest reliabilities are summarized in Table 3. The reliability of single task walking speed, cadence, step duration, and step length was “excellent” [32] (ICCs between 0.824–0.898 and SEMs between 0.03–5.2) and comparable to the reliability of dual task walking speed, cadence, step duration and step length (ICCs between 0.826–0.869 and SEMs between 0.1–5.38).

### 4.3. Validity

The mean values and standard deviations of the gait parameters of 29 evaluated patients at baseline are reported in Table 4 for their grouping. Planned contrasts showed that there was no significant effect on SDu, *V*, and cadence and a significant effect of grouping on step length performance. This latter parameter, however, showed a large effect [38]. The planned contrasts revealed that having mild and severe PN did not significantly alter step length compared to diabetic patients presenting without PN, *t*(26) = −1.318, *P* = .101, and having severe PN significantly influenced step length compared to mild PN, *t*(26) = −2.469, *P* = .046 (one tailed).

## 5. Discussion

This study has shown that the reliability of walking speed, cadence, step duration and step length on different surfaces and under dual task conditions was high with excellent ICCs, small SEMs and RLOAs in older adults with diabetes using the DynaPort MiniMod system. Results from discriminant validity were essentially non conclusive, with the exception of step length. There are, therefore, only indications that the system might also be able to distinguish between subpopulations within the population of patients with diabetes based on step length. The disease status of the elderly participants in our study varied from having diabetes without PN and having diabetes with mild or severe polyneuropathy. We thus expected our subjects to represent a heterogeneous group with regard to walking abilities. From previous studies we know that disease severity negatively influences walking velocity [4] especially in challenging environments where patients with neuropathy walk slower when compared to patients without neuropathy [12]. We think that the negative findings in our cross-sectional sample are very likely related to the limited statistical power of this analysis and might be attributed to a possible Type I error. A post hoc power analysis revealed that Power (1-*β* err prob) = 0.19. Our data allow for an a priori sample size calculation for a future trial with a fixed effects one-way ANOVA design and under the assumption of a moderate effect size of 0.25. To avoid a type I or II error in this future trial, we need an estimated sample size of 159 (53 individuals per group). This would result in 80% power at *α*-level 0.05 [39].

The gait changes that we observed in dual task walking relative to single task walking are consistent with other studies that demonstrate that cognitive tasks have a destabilizing effect on gait [40–44]. This finding seems to indicate that it is important to consider additional cognitive tasks in gait assessment of diabetic patient populations in clinical practice.

There is scarce information available about the reliability of body fixed sensor approaches to assess gait parameters in older adults with diabetes. The ICCs for walking speed and cadence that we found were, however, similar to values reported by Allet et al. [19] who were using the Physilog system in older, diabetic subjects.

The relative reliability is the degree to which individuals maintain their test results in a sample with repeated measurements and is affected by sample heterogeneity, that means the more heterogeneous a sample is, the higher the relative reliability becomes. Therefore, a high correlation may still mean unacceptable measurement error for some analytical goals, for example, for individualised assessments [36], and data about absolute reliabilities of a test are desired for clinical use. The determination of what constitutes an acceptable RLOA depends on what size difference the researcher or clinician wants to detect when comparing groups or when assessing the effect of interventions [45]. Whether the absolute reliability reported here for the gait measures is sufficiently high to identify gait impairments or small effects of an intervention program to improve walking in populations suffering from diabetes should be part of future studies. In particular, the RLOAs for step length and gait velocity might be indicative for rather large needed changes to be detected with the system. A study that investigated gait recovery in a sample of patients with diabetes due to specific exercises [46], with a mean age of 63 years and that used the Physilog gait analysis system for evaluation in challenging environments, showed that changes in gait velocity of around 0.149 m·s^−1^, and improvements of 10% for cadence are achievable with specific rehabilitation [5, 6]. Whether such changes are also clinically meaningful should be determined in future studies.

In the present study we have shown that step length measures derived from the DynaPort MiniMod are significantly different between groups of patients. There are measurable differences between individuals with mild and severe PN. Clinical detection of these differences potentially allows the division of diabetes patients into two groups with different mean step length: one with severe NP and one without severe NP. These results support the assertion that there is a relationship between quality of walking and the presence of PN. However, these are only preliminary data, and further (cross-sectional and longitudinal) research is needed with larger samples to substantiate this observation.

To obtain the diagnostic information from a walking test in a challenging environment alone, the outcomes of the gait analysis should be compared with other diagnostic tests in use. This necessitates the concurrent measurement of those tests in future research. Therefore, bigger samples of subjects should be selected in the future, and with logistic regression analysis the contribution of the DynaPort MiniMod gait assessment to existing diagnostic tests should be estimated more precisely [47].

## 6. Conclusions

The results of this study demonstrate that walking speed, cadence, step duration, and step length under more challenging conditions can be reliably measured in adults with diabetes using the DynaPort MiniMod system. There are first indications that the system is able to discriminate subgroups of patients with diabetes based on their step length. Further research in diabetic populations is needed to determine the value of these parameters that are derived from this measurement system in clinical settings.

## Figures and Tables

**Table 1:** Demographic description of the consecutively recruited subjects at baseline.

Subject (no.)	Sex (m/f)	Age (years)	Weight (kg)	Height (m)	BMI (kg/m^2^)	Group^∗^
01	m	60	82	1.76	26.5	2
02	m	70	88	1.74	29.1	2
03	f	64	92	1.73	30.7	3
04	m	63	94	1.76	30.4	3
05	m	69	84	1.76	27.1	2
06	m	57	102	1.78	32.2	2
07	f	63	67	1.55	27.9	2
08	m	70	95	1.76	30.7	3
09	m	69	73	1.79	22.8	2
10	m	57	98	1.84	29	3
11	m	61	103	1.79	32.2	1
12	m	69	66	1.72	22.3	2
13	m	53	90	1.71	30.8	3
14	m	70	102	1.85	29.8	3
15	f	67	78	1.64	29	1
16	m	65	94	1.74	31.1	1
17	m	62	81	1.75	26.5	1
18	m	62	92	1.70	31.8	1
19	m	56	108	1.82	32.6	1
20	m	59	67	1.67	24	1
21	m	60	76	1.73	25.4	1
22	m	56	93	1.70	32.2	1
23	f	64	54	1.60	21.1	2
24	m	60	92	1.78	29.0	1
25	f	50	60	1.63	22.6	2
26	m	57	85	1.80	26.2	2
27	f	60	75	1.60	29.3	1
28	m	55	85	1.78	26.8	1
29	m	65	81	1.87	23.2	2

BMI: body mass index; ^∗^1: “diabetic,” 2: “mild neuropathy,” and 3: “severe neuropathy”; m/f: male/female.

**Table 2:** Results of repeated measurements (*N* = 25).

	Test visit 1 mean ± SD	Test visit 2 mean ± SD	*P* value
Single task			
SDu (s)	0.54 (0.051)	0.55 (0.045)	0.771
SL (m)	0.69 (0.095)	0.69 (0.111)	0.884
*V* (m/s)	1.28 (0.213)	1.28 (0.243)	0.942
Cad (step/min)	111.7 (10.234)	111.0 (9.031)	0.652
Dual task			
SDu (s)	0.58 (0.079)	0.56 (0.056)	0.046
SL (m)	0.67 (0.109)	0.68 (0.107)	0.325
*V* (m/s)	1.17 (0.273)	1.23 (0.233)	0.154
Cad (step/min)	104.51 (12.626)	107.7 (10.170)	0.046

SD: standard deviation; SDu: step duration; SL: step length; *V*: velocity; Cad: cadence.

**Table 3:** Reliability of different gait parameters^a^ at preferred speed (ICC: intraclass correlation coefficient, CI 95% confidence interval 95%, SEM standard error of measurement, SDD smallest detectable difference, and LALB limits of agreement lower boundary, LAUB limits of agreement upper boundary).

	ICC	CI 95%	SEM	CI 95%	SDD	LALB	LAUB
Single task							
SDu (s)	0.848	0.652–0.933	0.03	±0.06	0.09	−0.072	0.071
SL (m)	0.898	0.767–0.955	0.04	±0.09	0.12	−0.128	0.124
*V* (m/s)	0.824	0.597–0.923	0.12	±0.25	0.35	−0.349	0.354
Cad (step/min)	0.834	0.623–0.927	5.20	±10.2	14.42	−13.754	15.096
Dual task							
SDu (s)	0.829	0.597–0.926	0.10	±0.20	0.28	−0.077	0.119
SL (m)	0.869	0.706–0.942	0.17	±0.34	0.48	−0.159	0.130
*V* (m/s)	0.829	0.616–0.924	0.13	±0.26	0.37	−0.431	0.318
Cad (step/min)	0.826	0.672–0.940	5.38	±10.54	14.90	−18.101	14.901

SDu: step duration; SL: step length; *V*: velocity; Cad: cadence.

^a^Calculations—SEM:$\sqrt{\text{Mean}}$ square error; CI 95% = ±1.96 × SEM; SDD = 1.96 × $\sqrt{2}$ × SEM.

**Table 4:** The mean and standard deviations of the gait parameters of 29 evaluated patients, grouped based on disease status, at baseline.

Performance measure		Group			
	Diabetic (*n* = 12)	Mild neuropathy (*n* = 11)	Severe neuropathy (*n* = 6)
Single task			
Step duration (SDu; s)	0.56 ± 0.03	0.53 ± 0.05	0.55 ± 0.05
Step length (SL; m)	0.72 ± 0.06	0.73 ± 0.1	0.62 ± 0.12
Velocity (m·s^-1^)	1.29 ± 0.14	1.39 ± 0.14	1.14 ± 0.37
Cadence (steps/min)	107 ± 5.8	115 ± 10.2	107.6 ± 15
Dual task			
Step duration (SDu; s)	0.6 ± 0.08	0.55 ± 0.05	0.6 ± 0.12
Step length (SL; m)	0.7 ± 0.07	0.7 ± 0.13	0.63 ± 0.1
Velocity (m·s^-1^)	1.18 ± 0.21	1.31 ± 0.28	1.09 ± 0.32
Cadence (steps/min)	101.4 ± 11	110 ± 9.3	103.4 ± 17.6
